# A Series of Anatomical Plates, in Lithography

**Published:** 1834-01-01

**Authors:** 


*A Series of Anatomical Plates, in Lithography; with References and Physiological Comments, illustrating the Structure of the different Parts of the Human Body.* Edited by Jones Quain, M.D., Professor of Anatomy and Physiology in the University of London.—1833. Fasciculi I—IV.

This work is to consist of 125 fasciculi, two of which are to make their appearance every month, and therefore, supposing the thing to go on with the most scrupulous exactitude, the book will be complete in five years, two months, and a fortnight. To give an opinion of a work with only $\frac{4}{125}$ of it before us, would be as rash as to pass judgment on a dinner, after having swallowed nothing but ℥ss. of soup; yet we may say that the eight plates in the fasciculi before us are good and cheap; and, should the remaining 242 be as well executed, we shall recommend our readers to buy the book.

